# Ectopic Expression of a Poplar Gene *PtrMYB119* Confers Enhanced Tolerance to Drought Stress in Transgenic *Nicotiana tabacum*

**DOI:** 10.3390/plants14213251

**Published:** 2025-10-23

**Authors:** Weibing Zhuang, Li Sun, Jiaqi An, Jie Zhu, Tengyue Yan, Tao Wang, Xiaochun Shu, Zhong Wang

**Affiliations:** 1 Jiangsu Key Laboratory for Conservation and Utilization of Plant Resources, Institute of Botany, Jiangsu Province and Chinese Academy of Sciences (Nanjing Botanical Garden Memorial Sun Yat-Sen), Nanjing 210014, China; 2 Lin Yi State Owned Holding Gardens Co., Ltd., Linyi 276000, China

**Keywords:** *PtrMYB119*, drought tolerance, antioxidant enzyme system, drought-responsive genes

## Abstract

Drought stress is a major limiting factor during the process of plant growth and development, especially in arid and semi-arid regions. MYB transcription factors play vital roles in the regulation of many developmental processes under various stresses. The aim of this study was to determine whether *PtrMYB119* enhanced dehydration tolerance in *Nicotiana tabacum*. *PtrMYB119*, with a weak transactivation activity, was distributed throughout the cell with no apparent specificity. The transgenic tobacco overexpressing *PtrMYB119* might regulate dehydration tolerance through increased ABA content and antioxidant enzyme activities, decreased MDA levels, and up-regulation of antioxidant genes, polyamine biosynthesis genes, and drought-responsive genes. Overall, our results could contribute to the elucidation of drought tolerance underlying *PtrMYB119* action in tobacco and indicated that *PtrMYB119* could be exploited for engineering drought-enduring plants in the future.

## 1. Introduction

As the global climate gets warmer and warmer, drought stress is becoming a great threat to future crop production and food security [1,2]. Up to now, more than one-third of the land around the world is arid land, and it is predicted that this will increase by up to two-thirds by 2050, which will cause serious plant growth problems and crop yield reduction [3,4]. Therefore, it is important to understand the mechanisms of drought tolerance in plants.

When plants are subjected to drought stress, many important morphological and physiological parameters undergo changes, such as leaf wilting, antioxidant enzyme system, endogenous abscisic acid (ABA), and so on [5]. To cope with drought conditions, plants undergo morphological adjustments, including modifications like diminished leaf area, augmented leaf thickness, elevated density of epidermal trichomes, enhanced leaf wax layer, decreased stomatal count, more robust palisade tissues, a greater proportion of palisade to spongy parenchyma thickness, and the development of more elaborate vascular sheaths [6]. Drought stress triggers the buildup of reactive oxygen species (ROS) within plants, a process that in turn stimulates the activity of protective enzymes, thereby mitigating the harm inflicted by ROS on cellular components [7]. Additionally, plant hormones such as ABA play crucial roles in perceiving and reacting to drought stress [8]. Drought stress signaling mechanisms can be categorized into ABA-dependent and ABA-independent regulatory pathways. Both pathways are activated when plants are exposed to drought and engage in distinct signal transduction cascades to counteract the stress [9]. Under drought conditions, the transcript levels of transcription factors (TFs) involved in various signaling pathways undergo changes [10]. MYB TFs are extensively present in higher plants and constitute the largest and most functionally diverse TF family, exerting a central role in plant stress tolerance [11]. A significant number of MYB genes are regulated by drought; for instance, in rice, 65% of drought-associated MYB genes are affected, and in *Arabidopsis*, 51% of AtMYB genes are up-regulated by drought, while 41% are down-regulated [12,13]. Under drought stress, MYB TFs participate in regulation through various mechanisms. For example, the poplar (*Populus tomentosa* Carr) transcription factor PtoMYB142 can directly bind to the promoters of the wax biosynthesis genes CER4 and KCS6, activating their transcription, which leads to increased leaf wax accumulation and significantly enhances drought tolerance in poplar [14]. In transgenic tomatoes, *SlMYB50* negatively regulates drought and high salt resistance by influencing chlorophyll synthesis, flavonoid synthesis, carotenoid synthesis, antioxidant enzymes, and ABA synthesis [15]. Furthermore, MdMYB88 and MdMYB124 in apple regulate root architecture and xylem development by directly binding to the promoters of *MdVND6* and *MdMYB46*, which are key regulators of secondary cell wall biosynthesis. This regulation improves the plant’s adaptation to drought [16], indicating that MYB TFs can contribute to plant drought stress responses by modifying root system architecture. The overexpression of *MdoMYB121* in tomato could enhance its drought tolerance partially by reducing the water loss [17]. The ectopic overexpression of *OsMYB4*, *OsMYB3R-2,* and *OsMYB2* from rice could facilitate its drought resistance through the constitutive activation of several stress-inducible pathways and different kinetics in the accumulation of several metabolites [18,19,20]. The overexpression of *TaMYB30-B* in Arabidopsis thaliana also improved its drought tolerance through the changes in physiological traits (water loss, proline, MDA, and soluble sugar contents) and the altered expression of drought stress-responsive genes (*RD29A* and *ERD1*) [21]. *VvMYB60*, a gene from grapevine with high similarity to *AtMYB60*, could increase its drought tolerance through the modulation of guard cells and the reduction in water loss [22]. The overexpression of *StMYB1R-1* in potato plants improved plant tolerance to drought stress through reduced rates of water loss, more rapid stomatal closing, and enhanced the expression of drought-regulated genes, such as *AtHB-7*, *RD28*, *ALDH22a1*, and *ERD1-like* [23]. The expression of *CmMYB2* in chrysanthemum leaves was up-regulated in response to drought, and the overexpression of *CmMYB2* in Arabidopsis thaliana enhanced their drought tolerance due to their reduced rates of water loss [24]. It has been reported that anthocyanins are important antioxidants, which can protect plants from adversity damage, and plants with high anthocyanin content also have high antioxidant capacity [25,26]. Some R2R3-MYB transcription factors were found to induce anthocyanin accumulation by regulating anthocyanin biosynthesis-related genes [27,28,29]. *SbMYB8*, a R2R3-MYB gene from *Scutellaria baicalensis*, was shown to regulate flavonoid biosynthesis, and the overexpression of which enhanced its drought stress resistance in transgenic tobacco [30]. Overexpression of the snapdragon Delila (*Del*) gene in tobacco enhanced anthocyanin accumulation, which improved their drought tolerance [31]. The aforementioned research demonstrated that many MYB transcription factors (TFs) have been shown to be involved in the responses to abiotic stresses, which could function in drought response and tolerance through the expression level of key components involved in photosynthesis, ABA and/or auxin signaling networks, as well as antioxidant biosynthesis for ROS scavenging and metabolic adjustments, including wax and flavonoid biosynthesis [32,33]. *PtrMYB119*, a R2R3-MYB transcription factor from *Populus trichocarpa*, and the overexpression of which could enhance anthocyanin production in hybrid poplar [34]. However, whether *PtrMYB119* could enhance drought tolerance in poplar and other plants is still unknown.

In the present study, our goal was to verify the roles of *PtrMYB119* in improving drought tolerance and to investigate the underlying mechanisms. Our data provided a comprehensive resource for further molecular research on this species, which could contribute to the elucidation of drought tolerance underlying *PtrMYB119* action in tobacco and also provided references for engineering drought-tolerant plants in the future.

## 2. Results

### 2.1. PtrMYB119 Was Responsive to the Drought Treatment

To explore the expression patterns of *PtrMYB119* in poplar under drought stress, the expression levels of *PtrMYB119* in poplar were determined after drought treatment. After 7 days of drought treatment, the expression levels of *PtrMYB119* in poplar increased significantly compared with the 0 days of drought treatment (Supplementary Figure S1), which indicated that *PtrMYB119* might be involved in the drought response in poplar.

### 2.2. PtrMYB119 Was Localized to Nucleus and Cytoplasm with a Weak Transactivation Activity

**Figure 1:**
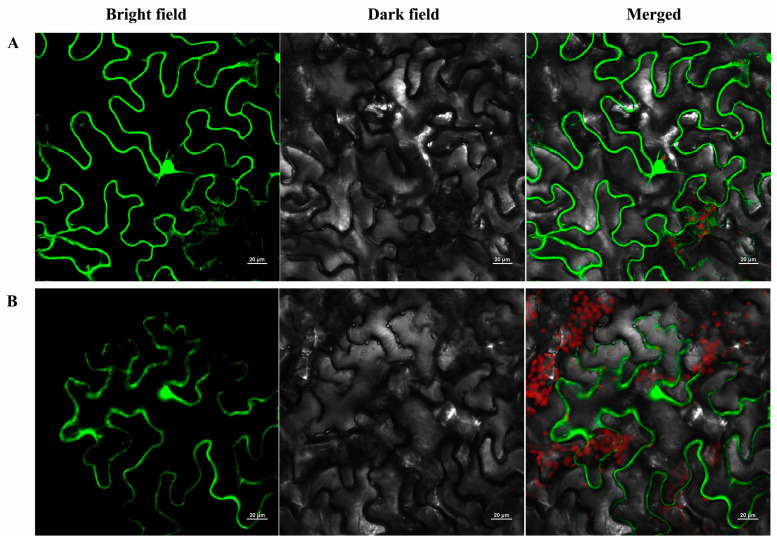
Subcellular localization analysis of *PtrMYB119* protein. (**A**): p35S-GFP, (**B**): p35S-PtrMYB119-GFP.

To explore the subcellular localization of *PtrMYB119*, the full-length ORF of *PtrMYB119* was fused to the N-terminal of GFP reporter protein to generate a fusion protein *PtrMYB119*: GFP, which was driven by the CaMV35S promoter, and the plasmid containing GFP alone was used as a control. The fusion protein (*PtrMYB119*) and the control (GFP) were analyzed in tobacco leaf epidermis with a confocal laser scanning microscope. The control GFP was uniformly distributed throughout the whole cell (Figure 1A), whereas the *PtrMYB119*-GFP fusion protein was also observed in the nucleus and cytoplasm (Figure 1B), indicating that *PtrMYB119* was distributed throughout the cell with no apparent specificity.

**Figure 2:**
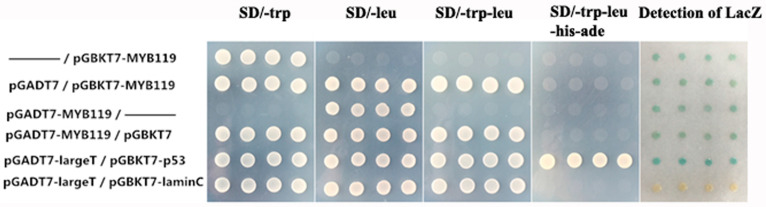
The transcriptional activation ability of *PtrMYB119* was examined using the yeast one-hybrid assay. Yeast cells Y2H expressing the fusion proteins were cultured and adjusted to 2.0 (OD600), and then series diluted and dropped with 2 mL on nutritional selective medium (SD/-trp, SD/-leu, SD/-trp-leu, SD/-trp-leu-his-ade). Yeast cells expressing pGADT7-large T/pGBKT7-lamin C served as the negative control and pGADT7-large T/pGBKT7-p53 as the positive control. The signals of other samples are much weaker compared with those in the p53 positive control, and stronger than those in the negative control. Photos were taken after incubating at 30 °C for 2–4 d.

In addition to subcellular localization, transactivation activity is another defining feature of a transcription factor. The Y2H system was used to investigate whether *PtrMYB119* functioned as a transcriptional activator. For this purpose, the coding sequence of *PtrMYB119* was fused to the coding sequence of *GAL4* to generate a fusion plasmid, which was transformed into yeast AH109 to see the growth status of cells on the nutritional selective medium. The positive control was pGADT7-large T/pGBKT7-p53, which showed better growth status of cells on the four nutritional selective medium (SD/-trp, SD/-leu, SD/-trp-leu, SD/-trp-leu-his-ade) and had blue colonies; the negative control was pGADT7-large T/pGBKT7-lamin C, which can grow well on the three nutritional selective media (SD/-trp, SD/-leu, SD/-trp-leu), and cannot grow on the nutritional selective medium of SD/-trp-leu-his-ade (Figure 2). The negative control cannot produce blue colonies compared with the positive control. There were slight blue colonies for the vectors containing *PtrMYB119* compared with the positive control, indicating that *PtrMYB119* had a weak transactivation activity. On the nutritional selective medium of SD/-trp-leu-his-ade, transgenic yeast cells harboring pGBKT7-MYB119, pGADT7-MYB119, pGADT7/pGBKT7-MYB119, or pGADT7-MYB119/pGBKT7 were able to grow; however, the positive control grew better compared with these transgenic yeast cells (Figure 2). Taken together, these results demonstrated that *PtrMYB119* had a weak transactivation activity in yeast cells.

### 2.3. The Expression Patterns of PtrMYB119 in Transgenic Tobacco Under Drought Treatments

**Figure 3:**
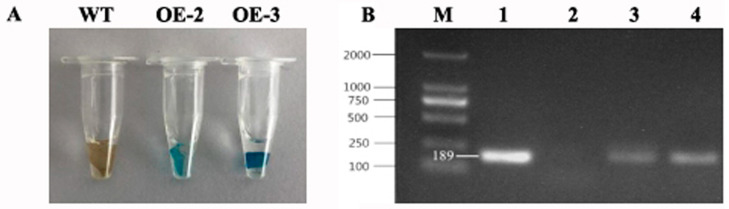
(**A**) GUS staining of transgenic plants overexpressing *PtrMYB119* (OE-1, transgenic OE-1 line; OE-2, transgenic OE-2 line; OE-3, transgenic OE-3 line). (**B**) PCR detection of transgenic plants overexpressing *PtrMYB119* (M, marker; 1, *PtrMYB119* vector; 2, WT; 3, transgenic OE-2 line; 4, transgenic OE-3 line).

Transgenic tobacco overexpressing *PtrMYB119* was generated to investigate the functions of *PtrMYB119* under drought stress. GUS staining and genomic PCR were used to detect whether they were transgenic plants. As shown in Figure 3A, the results of GUS staining indicated that transgenic lines OE-2 and OE-3 were positive. The putative *PtrMYB119* fragment (186 bp) with special primers was also amplified from these transgenic lines, OE-2 and OE-3 (Figure 3B).

**Figure 4:**
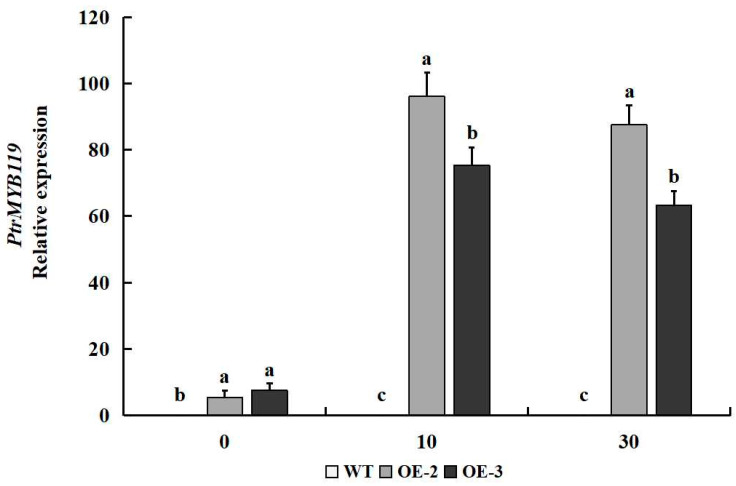
Expression level of *PtrMYB119* in transgenic plants and WT plants after 0, 10, and 30 days of drought treatment. Data are means ± SE of three biological replicates, and means followed by different letters are significantly different (*p* < 0.05). The X-axis is the days of drought treatment (OE-2, transgenic OE-2 line; OE-3, transgenic OE-3 line).

The real-time quantitative PCR (qPCR) was performed to evaluate the time-course expression levels of *PtrMYB119* in leaves of transgenic tobacco under drought treatment. After 10 days of drought treatment, the expression levels of *PtrMYB119* in leaves of transgenic tobacco increased sharply, which indicated that *PtrMYB119* in transgenic tobacco might play crucial roles under drought treatment. After 30 days of drought treatment, the expression levels of *PtrMYB119* in leaves of transgenic tobacco had a slight decrease, but were still much higher than those on 0 days of drought treatment (Figure 4). Similarly, there was no expression level of PtrMYB119 in WT plants after 10 days and 30 days of drought treatment (Figure 4).

### 2.4. Overexpression of PtrMYB119 Affects the Growth of Tobacco Plants Under Drought Stress

**Figure 5:**
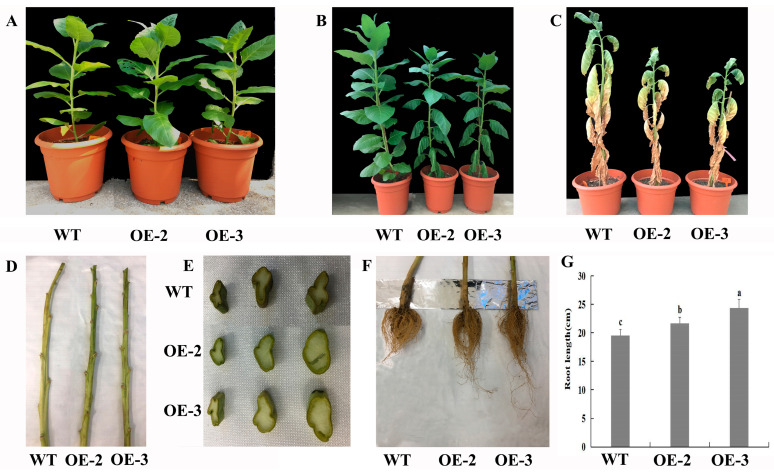
(**A**) Transgenic and WT tobacco before drought treatment. (**B**) Growth of transgenic and WT lines after 10 days of drought treatment. (**C**) Growth of transgenic and WT lines after 30 days of drought treatment. (**D**) Stem after 30 days of drought treatment. (**E**) Cross-section of the same part of the stem of transgenic and WT lines. Data are means ± SE of three biological replicates, and means followed by different letters are significantly different (*p* < 0.05). (**F**) Root growth after 30 days of drought treatment. (**G**) Root length after 30 days of drought treatment (OE-1, transgenic OE-1 line; OE-2, transgenic OE-2 line; OE-3, transgenic OE-3 line).

The leaves of lines OE-2 and OE-3 appeared wilted first after 10 days of drought treatment, whereas leaves of WT grew normally, which indicated that lines OE-2 and OE-3 responded to drought stress earlier (Figure 5A,B). After 30 days of drought treatment, there were many more withered leaves and a higher ratio of withered leaves to total leaves in WT plants compared with those in lines OE-2 and OE-3 (Figure 5C, Supplementary Figure S2). After the leaves of transgenic and WT plants were removed, the branches of lines OE-2 and OE-3 were much greener, and the degree of wilting and shrinkage in them was less severe compared with those in WT plants (Figure 5D). The cross-sections of the same stem part in transgenic tobacco and WT plants were evaluated, and the results showed that the cross-section of stems in WT plants were more severely wilted and had severe shrinkage compared with those in transgenic tobacco, and the color of the cross-section stem in WT plants was dark compared with those in the transgenic tobacco, indicating that transgenic plants might have better drought tolerance compared with WT plants (Figure 5E). The stem diameters of lines OE-2 and OE-3 were significantly higher than those of WT plants after 30 days of drought treatment (Supplementary Figure S3). However, the plant heights of lines OE-2 and OE-3 were significantly lower than those of WT plants after 30 days of drought treatment (Supplementary Figure S4). To better understand the functions of *PtrMYB119* on the growth of transgenic tobacco, the fresh weight and dry weight were evaluated. Although the transgenic lines OE-2 and OE-3 were shorter than WT plants, they had higher fresh weights and dry weights than WT plants after 30 days of drought treatment (Supplementary Figure S5). In addition, the mean root lengths of OE-2 and OE-3 plants were 21.7 ± 1.0 and 24.4 ± 1.5 cm after 30 days of drought treatment, while that of WT plants was 19.5 ± 1.1 cm, which showed that transgenic tobacco lines OE-2 and OE-3 had significantly longer roots (Figure 5F,G), indicating that there was a possible trade-off between growth and stress tolerance in transgenic plants. Overall, transgenic tobacco plants overexpressing *PtrMYB119* responded to drought stress more rapidly than WT plants after 10 days of drought treatment, and showed better drought tolerance after 30 days of drought treatment due to their phenotype and physiological indicators.

### 2.5. The Content of Anthocyanin and Chlorophyll in Tobacco Plants Overexpressing PtrMYB119 Under Drought Stress

**Figure 6:**
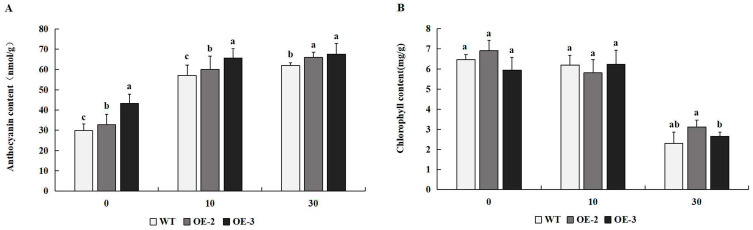
(**A**) Anthocyanin content in transgenic and WT plants after drought treatment. (**B**) Chlorophyll content in transgenic and WT plants after drought treatment. Data are means ± SE of three biological replicates, and means followed by different letters are significantly different (*p* < 0.05). The X-axis is the days of drought treatment (OE-2, transgenic OE-2 line; OE-3, transgenic OE-3 line).

The content of anthocyanin in transgenic tobacco overexpressing *PtrMYB119* was higher than that in WT plants after 0 days of drought treatment, especially in the transgenic line OE-3. After 10 days and 30 days of drought treatment, the transgenic plants had higher anthocyanin content compared with that in WT plants (Figure 6A). There was no significant difference in chlorophyll content between WT and transgenic tobacco overexpressing *PtrMYB119* after 0 days and 10 days of drought treatment. However, chlorophyll content decreased similarly in WT and transgenic plants under prolonged drought (Figure 6B).

### 2.6. Overexpression of PtrMYB119 Affects the ABA Concentration of Tobacco Plants Under Drought Stress

**Figure 7:**
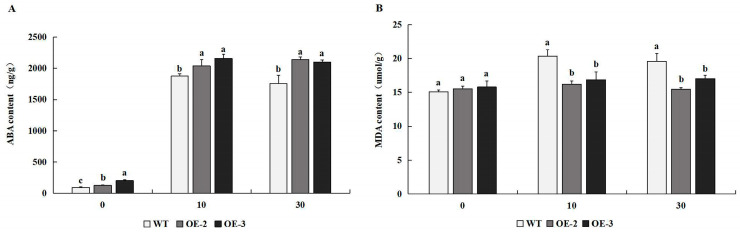
(**A**) ABA concentration in transgenic and WT plants after drought treatment. (**B**) MDA content in transgenic and WT plants after drought treatment. Data are means ± SE of three biological replicates, and means followed by different letters are significantly different (*p* < 0.05). The X-axis is the days of drought treatment (OE-2, transgenic OE-2 line; OE-3, transgenic OE-3 line).

The concentration of ABA in transgenic tobacco overexpressing *PtrMYB119* was significantly higher than that in WT plants (Figure 7A). After 10 days of drought treatment, there was a significantly greater increase in ABA concentrations in lines OE-2 and OE-3 than in WT. Compared with the ABA concentration in transgenic tobacco and WT at 10 days of drought treatment, there was less change in ABA concentration in transgenic tobacco and WT at 30 days of drought treatment (Figure 7A). After 10 and 30 days of drought treatment, ABA concentrations in transgenic tobacco and WT plants increased significantly, and ABA concentrations in transgenic tobacco were still much higher than those in WT plants, which indicated that drought stress induced the increase in ABA concentration.

### 2.7. Overexpression of PtrMYB119 Decreases MDA Contents of Tobacco Plants Under Drought Stress

MDA played important roles in plants under drought stress. There was no significant difference in MDA content among transgenic plants overexpressing *PtrMYB119* and WT plants at 0 days of drought treatment (Figure 7B). After 10 and 30 days of drought treatment, there was a significant difference in MDA content between the transgenic plants and WT plants, and the MDA content in transgenic plants was much lower than that in WT plants, which indicated that transgenic tobacco overexpressing *PtrMYB119* might enhance its drought tolerance through a regulatory effect on MDA content.

### 2.8. Overexpression of PtrMYB119 Enhances Antioxidant Enzyme Activities in Transgenic Tobacco Under Drought Stress

**Figure 8:**
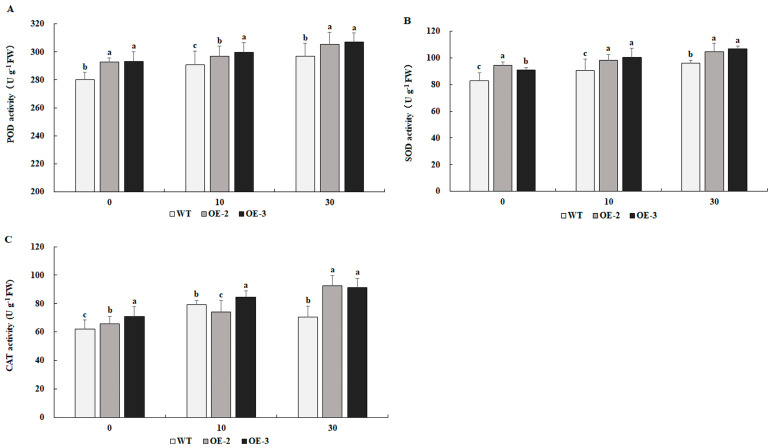
Antioxidant enzyme activities in transgenic and WT plants under drought treatment. (**A**) POD, (**B**) SOD, (**C**) CAT. Data are means ± SE of three biological replicates, and means followed by different letters are significantly different (*p* < 0.05). The X-axis is the days of drought treatment (OE-2, transgenic OE-2 line; OE-3, transgenic OE-3 line).

As shown in Figure 8A–C, transgenic tobacco overexpressing *PtrMYB119* showed higher POD, SOD, and CAT activities compared with those in WT plants after 0 days of drought treatment. After 10 days of drought treatment, POD and SOD activities increased significantly in both transgenic and WT plants; further, those activities were much higher in transgenic plants than in WT plants. After 30 days of drought treatment, the activities of POD and SOD increased significantly in both transgenic tobacco and WT plants, and they remained much higher in transgenic plants than these in WT plants. CAT activity increased after 10 and 30 days of drought treatment in transgenic plants, while the activity of CAT in WT plants increased after 10 days of drought treatment and decreased after 30 days of drought treatment.

### 2.9. Expression Levels of Drought Stress-Responsive Genes in Transgenic Tobacco Under Drought Stress

**Figure 9:**
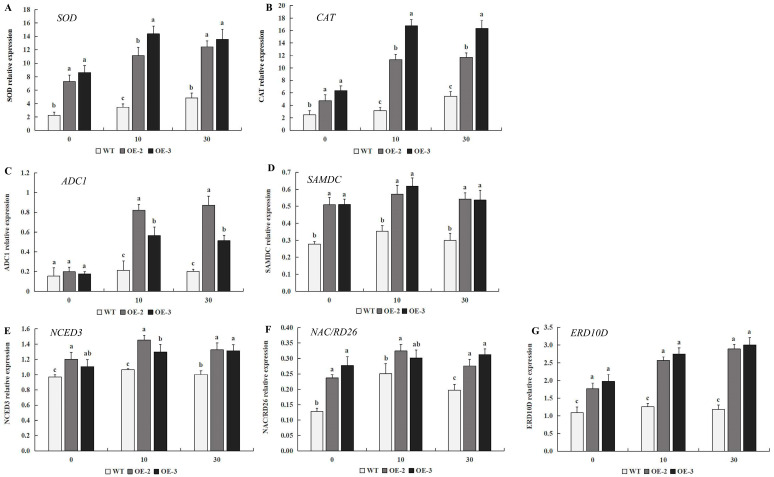
Expression levels of drought-related genes in transgenic and WT plants under drought stress. (**A**) Antioxidant enzyme-coding genes *SOD*. (**B**) Antioxidant enzyme-coding genes *CAT*. (**C**) Polyamine biosynthesis genes *ADC1*. (**D**) Polyamine biosynthesis genes *SAMDC*. (**E**) Drought-responsive genes *NCED3*. (**F**) Drought-responsive genes *NAC*/*RD26*. (**G**) Drought-responsive genes *ERD10D*. Data are means ± SE of three biological replicates, and means followed by different letters are significantly different (*p* < 0.05). The X-axis is the days of drought treatment (OE-2, transgenic OE-2 line; OE-3, transgenic OE-3 line).

To further investigate the gene expression pattern of transgenic tobacco overexpressing *PtrMYB119* under drought stress, transcript abundance of genes involved in drought stress was evaluated in transgenic plants and WT plants under drought conditions. As shown in Figure 9A,B, the relative expression levels of genes associated with the antioxidant enzyme system (*SOD* and *CAT*) in the transgenic plants were much higher than those in WT plants, which was consistent with the activity changes in antioxidant enzymes. Arginine decarboxylase 1 (*ADC1*) and S-adenosylmethionine decarboxylase (*SAMDC*), involved in the polyamine biosynthesis, can play important roles in various abiotic stresses, including drought stress. There was no significant difference in the expression level of *ADC1* between transgenic plants and WT plants at 0 days of drought treatment (Figure 9C). However, there was a significantly greater increase in *ADC1* expression level in transgenic plants compared with that in WT plants after 10 days of drought treatment, and the expression level of *ADC1* in transgenic plants was maintained at a high level after 30 days of drought treatment (Figure 9C). There is an increase in the wildtype but only a slight one compared to the increase in the transgenic lines after 10 days and 30 days of drought treatment (Figure 9C). Different from the expression level of *ADC1*, the expression levels of *SAMDC* in transgenic plants were much higher than those in WT plants after 0 days of drought treatment. After 10 days of drought treatment, the expression level of *SAMDC* in both transgenic plants and WT plants had a slight increase, and the expression levels of *SAMDC* in transgenic plants were still much higher than those in WT plants. After 30 days of drought treatment, the expression level of *SAMDC* in both transgenic plants and WT plants had a slight decrease, and the expression levels of *SAMDC* in transgenic plants were also much higher than those in WT plants (Figure 9D). These results indicated that the genes *SAMDC* and *ADC1* might play essential roles in the regulation of drought stress in tobacco. To better understand the molecular mechanism of drought tolerance in tobacco mediated by *PtrMYB119*, the expression levels of drought-responsive genes, including *ERD10D*, *NCED3,* and *NAC*/*RD26*, were evaluated. There was a slight increase in expression level for *NCED3* in both transgenic plants and WT plants after 10 days of drought treatment, and the expression level of this gene in transgenic plants is higher than that in WT plants. After 30 days of drought treatment, the expression level of *NCED3* in both transgenic plants and WT plants had a slight decrease, and the expression level of this gene in transgenic plants is still higher than that in WT plants (Figure 9E). The expression level of *NAC*/*RD26* in transgenic plants was much higher than that in WT plants after 0 days of drought treatment, and the increase in expression level for *NAC*/*RD26* in WT plants was much higher than that in transgenic plants after 10 days of drought treatment (Figure 9). After 30 days of drought treatment, the expression level of *NAC*/*RD26* in transgenic lines OE-2 and WT plants had a slight decrease, while the expression level of *NAC*/*RD26* in transgenic lines OE-3 had a slight increase (Figure 9F). For the gene *ERD10D*, its expression level in transgenic plants had a continued increase after 10 days and 30 days of drought treatment, while there was a low expression level in WT plants after 10 days and 30 days of drought treatment. The expression level of *ERD10D* in transgenic plants was always much higher than that in WT plants (Figure 9G). Overall, these results indicated that overexpression of *PtrMYB119* in transgenic plants enhanced drought tolerance by regulating the expression level of antioxidant genes, polyamine biosynthesis genes, and drought-responsive genes.

## 3. Discussion

Drought is a major abiotic stress that negatively affects vegetative and reproductive development of plants and reduces plant productivity. It is of great significance to discover the candidate genes associated with drought resistance to help plants cope with drought stress. Many MYB family genes have been responsive to drought stress when plants encounter drought stress. Among them, a few MYB genes have been identified and characterized in poplar. Although many reports have described the function of MYB TFs in drought stress of different plant species [35,36,37], it is still unclear and needs to be further explored.

Anthocyanins have been proven to be involved in drought tolerance in many plant species [30,31,38,39,40]. As an antioxidant, anthocyanins containing high contents of enzymatic and non-enzymatic antioxidants can scavenge excess ROS [41], the accumulation of which in the vacuoles prevents the overproduction of ROS due to abiotic stresses [42], which makes them osmo-regulators that maintain water homeostasis in plants [26]. Many researchers indicated that transgenic plants overexpressing MYB TFs increased anthocyanin content [30,32,43], and anthocyanin accumulation had been associated with drought tolerance in many plant species [30,31,38,39,40]. As the previous study indicated that the overexpression of *PtrMYB119* in hybrid poplar could promote the production of anthocyanin [34], a substantially increased anthocyanin content in transgenic lines was expected. In our study, the increasing trends of anthocyanin content in WT plants and transgenic plants were similar (Figure 6A), indicating that higher anthocyanin content might not be the main reason why transgenic tobacco overexpressing *PtrMYB119* showed higher drought tolerance.

MYB genes functioned with ABA and regulated plant response to drought stress [44,45,46,47]. The higher accumulation of the endogenous ABA content in transgenic plants could enhance their drought tolerance through stress-induced ABA accumulation [44,48]. *NCED* (9-cis epoxycarotenoid dioxygenase), a rate-limiting enzyme gene, played crucial roles in drought stress-inducible ABA biosynthesis and catabolism [49]. The transgenic *Arabidopsis thaliana* overexpressing *OsNCED3* could increase its ABA content dramatically to cope with various stresses. In accordance with a previous study, transgenic plants overexpressing *PtrMYB119* had a higher ABA accumulation compared with these in WT plants under drought treatment, and the expression levels of *NCED3* in transgenic tobacco overexpressing *PtrMYB119* were higher than those in WT plants (Figure 7A and Figure 9E), which indicated that *PtrMYB119* might play a positive role in drought tolerance in tobacco by regulating stress-induced ABA synthesis.

The accumulation of ROS induced by drought could lead to cell toxicity, membrane peroxidation, and even cell death [50,51]. In order to detoxify ROS accumulation, plants have evolved efficient enzymatic antioxidant defense systems, including SOD, POD, CAT, and GST. Changes in antioxidant enzyme activities have been widely reported in plants in response to drought stress [20,52,53], and plants with high levels of these antioxidant enzymes showed tolerance to drought, salinity, or oxidative stress [54,55,56]. Consistent with previous results, transgenic plants overexpressing *PtrMYB119* had higher activities of antioxidant enzymes (SOD and CAT) compared with WT plants, which also have higher expression levels of the two genes *SOD* and *CAT* (Figure 8 and Figure 9A,B). ROS accumulation can also lead to peroxidation of membrane lipids, which produces a mass of degradation products, such as MDA [57]. As a marker for lipid peroxidation, low MDA content in leaves has been linked to high drought tolerance [39,58]. MDA in *OsMYB2*-overexpressing plants was markedly lower than that in WT plants under abiotic stress [20]. The overexpression of both *GbMYB5* and *Rosea1* in tobacco had a lower MDA accumulation compared with WT plants, which contributed to enhanced drought tolerance in transgenic tobacco [59,60]. In this study, the MDA content was lower in the transgenic tobacco plants overexpressing *PtrMYB119* than that in WT plants under drought treatment (Figure 7B), which was consistent with previous results [20,59,60]. Therefore, *PtrMYB119* might contribute to enhanced drought tolerance in tobacco through scavenging ROS accumulation under drought stress.

Plants adapt to drought stress through the up-regulation of stress-responsive genes, and the overexpression of some MYB TFs in plants can enhance their drought tolerance via this mechanism [21,37,59,60,61,62]. *ERD10* (*C*/*D*) encoded group 5 LEA proteins, which had crucial roles in withstanding cellular dehydration. The high expression of *ERD10* (*C*/*D*) can provide more chaperones or protective proteins for maintaining membrane integrity to sustain plant growth during drought [63,64]. *NAC*/*RD26*, a drought-inducible gene encoding an NAC transcription factor, had pivotal roles in plants under drought treatment [59]. In this study, the expression of *ERD10D* and *NAC*/*RD26* was much higher in transgenic plants overexpressing *PtrMYB119* under drought stress (Figure 9), indicating that transgenic plants might enhance their drought tolerance through the regulation of drought stress-responsive genes. In addition, the accumulation of osmolytes such as proline and (poly)amine was critical to maintain plant cell turgor and cell structure stabilization during drought stress [8,49,65]. The genes *ADC1* and *SAMDC* participated in the biosynthesis of the osmo-protectants proline and polyamine, which function in resisting adverse environments by adjusting osmotic balance and protecting plasma membrane integrity [8]. There were higher expression levels of *ADC1* and *SAMDC* in transgenic plants overexpressing *PtrMYB119* under drought stress (Figure 9C,D), which agreed with previous results [37,59], indicating that the transgenic *PtrMYB119* plants might synthesize osmolytes to alleviate cellular damage when they were subjected to drought stress. The above results indicated that the overexpression of *PtrMYB119* in tobacco can enhance its drought tolerance by the up-regulation of stress-responsive genes.

## 4. Materials and Methods

### 4.1. Plant Materials and Reagents

Tobacco (*Nicotiana tabacum* L.) cultivar K326 was used as experimental material. *E. coli* strain DH5α and *Agrobacterium tumefaciens* EHA105 were purchased from Shanghai Weidi Biotechnology Co., Ltd. (Shanghai, China). The primers were synthesized by Nanjing Genscript Biotech (Nanjing, China).

### 4.2. Plasmid Construction

Total RNA was extracted from leaves of *Populus trichocarpa*. First-strand cDNA was synthesized using a first-strand cDNA synthesis kit (Vazyme: R233-01, Nanjing, China). Gene-specific primers of *PtrMYB119* were designed using Primer 5 software. The primer sequences are listed in Supplementary Table S1. PCR conditions were: 94 °C for 3 min, 30 cycles of 94 °C for 30 s, 60 °C for 30 s, 72 °C for 1 min, and finally 72 °C for 10 min. The PCR product and pCAMBIA2300 vector were digested by Sal I and Xbal, and the target gene fragments and the backbone of the pCAMBIA2300 vector were ligated with T4 DNA ligase to obtain the vector pCAMBIA2300-*PtrMYB119*, which was confirmed by double digestion with a restriction enzyme and sequencing. The constitutive expression system included the CaMV35S promoter, nopaline synthase (NOS) terminator system, coding sequence of *PtrMYB119*, β-glucuronidase gene (GUS), and kanamycin-resistant gene in the recombined vector pCAMBIA2300-*PtrMYB119* (Supplementary Table S2).

### 4.3. Transactivitional Activity and Subcellular Localization of PtrMYB119

For the transactivation assay, the complete ORF of *PtrMYB119* was amplified by PCR using primers (Supplementary Table S1) containing Sfi I restriction sites, and the amplicon was digested by Sfi I. The resultant fragment containing *PtrMYB119* was then fused downstream of the yeast GAL4 DNA-binding domain in pGBKT7 by recombination reactions. The fusion vector and the negative control (pGBKT7) were independently expressed in yeast strain AH109 according to the manufacturer’s instructions.

The full-length cDNA of the *PtrMYB119* was synthesized by Nanjing Zoonbio Tech, Co., Ltd. (Nanjing, China) and inserted into a polylinker site of the binary vector pCAMBIA1302 to generate a fusion construct (p35S-*PtrMYB119*-GFP). After sequence verification, the fusion construct (p35S-*PtrMYB119*-GFP) and the control vector (pCAMBIA1302) were transferred into *Agrobacterium tumefaciens* strain GV3101 by heat shock. The abaxial surfaces of 5-week-old *N. benthamiana* leaves were agroinfiltration with the bacterial suspension (OD600 = 0.5) and then kept in an incubator for 24 h, followed by live cell imaging under an inverted confocal microscope (Zeiss LSM 780, Oberkochen, Germany). The images were presented with bright field, dark field, and a merge of bright field.

### 4.4. Transformation of Transgenic Tobacco Plant

The pCAMBIA2300-*PtrMYB119* was transformed into *Agrobacterium tumefaciens* EHA105, and the plasmid was also confirmed by double digestion with a restriction enzyme and sequencing. Transgenic tobacco plants were obtained using the *Agrobacterium*-mediated method as previously described [66]. Transgenic tobacco plants were selected on MS medium containing 50 mg·L^−1^ Kanamycin (Km) and 100 mg·L^−1^ Timentin (Tim). Positive transgenic T_0_ plants from regenerated Km-resistant plants were screened through GUS staining and PCR detection. Wildtype (WT) tobacco plants were used as controls.

### 4.5. Drought Stress Treatment

For drought treatment, three lines were selected from transgenic plants overexpressing *PtrMYB119*, and WT plants were used as the control. Each line contained twenty-five plants to conduct the drought treatment. Firstly, tissue culture seedlings were grown for 20 days in the tissue culture room. Secondly, they were placed in a growth chamber for adaptation to grow better. After that, they were transplanted into a peat–perlite mixture (1:5 *v*/*v*) in plastic pots (30 × 25 × 22 cm) in the glass greenhouse at 20–25 °C during the day, 10–15 °C at night, and with a 12/12 h (day/night) photoperiod. Plants were irrigated every 3 days until treatment initiation. To conduct the drought treatment, more than eighteen plants of each line and WT plants with a similar phenotype, such as leaf size and plant height, under well-watered conditions were selected. The growth status of tobacco plants was observed and recorded every day. The plants were photographed and sampled every 10 days for up to 30 days.

The one-year *Populus* sp. Linn. ‘2025’ in the greenhouse was used to conduct the drought treatment. The leaves from five *Populus* sp. Linn. ‘2025’ were obtained after 0 days of drought treatment and 7 days of drought treatment, separately. Each data point was the average value of three replicates.

### 4.6. Measurement of Anthocyanin, Chlorophyll, MDA and POD, SOD and CAT Enzyme Activities

To explore the effects of physiological changes caused by the overexpression of *PtrMYB119* in transgenic tobacco plants under drought treatment, similar leaves in each biological replicate were collected from three random transgenic and WT tobacco plants under drought treatment to determine the content of anthocyanin, chlorophyll, malondialdehyde (MDA), and the activities of peroxidase (POD), superoxide dismutase (SOD), and catalase (CAT). Total anthocyanin and chlorophyll contents were evaluated with previous methods [67]. The POD, SOD, and CAT activities and MDA content were determined using previous methods [59]. Each data point was the average value of three replicates.

### 4.7. Measurement of Endogenous ABA Content

All reagents and ABA were obtained from Sigma-Aldrich. HPLC-grade methanol, acetone, glacial acetic acid, formic acid, and acetonitrile were purchased from Merck. Fresh leaves (1 g) of transgenic and WT plants were ground in an ice-cold mortar and extracted twice with acetone/water/acetic acid (80:19:1, *v*/*v*/*v*) at −20 °C. After centrifugation, the extract from the second extraction was reconstituted in 200 μL of acetonitrile/water/acetic acid (90:10:0.05, *v*/*v*/*v*). The final extract was filtered and added to the LC-MS/MS system. Quantification was finished by the standard addition method by spiking control samples with ABA solutions [68]. Each data point was the average value of three replicates.

### 4.8. Quantitative Real-Time PCR

Total RNA was extracted according to the manufacturer’s instructions, and then the extracted RNA was reverse transcribed using a PrimeScript™1st Strand cDNA Synthesis Kit according to the kit instructions. Quantitative real-time PCR was performed using TB Green™ Premix Ex Taq™ II (Tli RNaseH Plus) (TaKaRa, Dalian, China). Each 20 µL quantitative real-time PCR contained 10 µL of TB Green^TM^ PCR master mix, 0.2 mM of each primer and 10 ng of cDNA with the following PCR program, 95 °C for 5 min, followed by 40 cycles of 95 °C for 15 s, and 62 °C for 1 min in an ABI 7300 Real-Time PCR system (Applied Biosystems, Foster City, CA, USA). All results were calculated from three biological replicates with three technical replicates. Drought stress-responsive genes in tobacco plants were analyzed at 10 and 30 d after drought treatment initiation. *NtTubulin* (N181029A17) was used as a house-keeping gene to investigate gene expression in transgenic tobacco plants overexpressing *PtrMYB119* and WT plants. The leaves of *Populus* sp. Linn. ‘2025’ after 0 days and 7 days of drought treatment were collected. *UBQ* (Accession number: AF240445) was used to normalize gene expression in poplar [67]. The UBQ primers for qRT-PCR were the following: UBQ-F (5′-TGAACCAAATGATACCATTGATAG-3′) and UBQ-R (5′-GTAGTCGCGAGCTGTCTTG-3′). All gene-specific primers were designed with Primer 5 software and are listed in Supplementary Table S3. The relative abundance of the genes was determined by the method [69].

### 4.9. Statistical Analysis

The experiments were repeated three times with three technical replicates. All data were expressed as mean ± standard error of three biological replicates. Differences among means of the various treatments were determined by the least significant difference test. Significance analysis was performed using SPSS 17.0 software. Normal distribution was tested with the Shapiro–Wilk test, and homogeneity of variance was tested with Levene’s test. One-way analysis of variance (ANOVA) was carried out to test the significance of treatments at *p* < 0.05, followed by Duncan’s tests. Means were considered to be significantly different when *p* ≤ 0.05, as shown in the figures. Microsoft Excel and GraphPad Prism 5.0 software were used for data analysis and charting.

## 5. Conclusions

**Figure 10:**
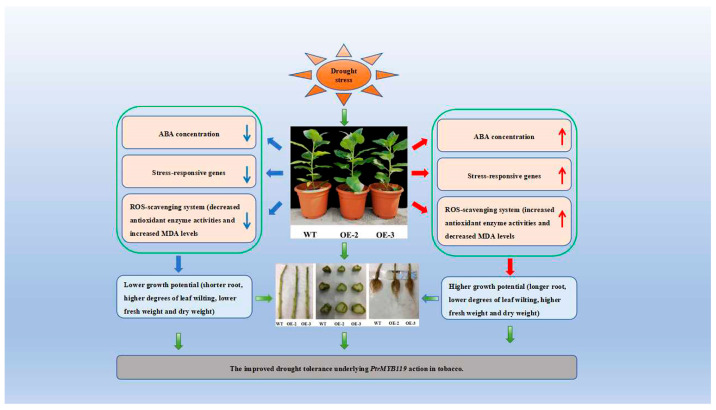
Proposed mechanisms of *PtrMYB119* enhanced drought tolerance in tobacco.

In summary, *PtrMYB119* overexpression lines showed elevated ABA content, antioxidant enzyme activity, drought stress-responsive gene expression levels, and reduced MDA content following drought treatment, suggesting that *PtrMYB119* modulated tolerance to drought in tobacco (Figure 10). The results of this study indicated that *PtrMYB119* played an important role in response to drought stress in tobacco and may be a potential gene for improving drought tolerance in other plants. In addition, the drought tolerance function of the *PtrMYB119* gene has only been validated in tobacco, and further validation is needed in other woody plants such as poplar trees.

## Data Availability

All data analyzed in this study are included in this published article and Supplementary Materials.

## Supplementary Materials

The following supporting information can be downloaded at: https://www.mdpi.com/article/10.3390/plants14213251/s1.
