# Supplementary material for: Ectopic Expression of a Poplar Gene PtrMYB119 Confers Enhanced Tolerance to Drought Stress in Transgenic Nicotiana tabacum
Source: Plants (Basel). 2025 Oct 23;14(21):3251. doi: 10.3390/plants14213251 (PMC12608618; doi:10.3390/plants14213251)
Supplement: Supplementary file 1 [file plants-14-03251-s001.zip › plants-3890811-supplementary.pdf]

## SUPPLEMENTARY MATERIALS

Supplementary Table S1. Primers used in PCR analysis in the present study

| Primer | Sequence (from 5' to 3) | Usage |
|---|---|---|
| PrtMYB119-F | ACGC gtcgacATGGTAGGCTCATTAGGAGT | Use for the plasmid construction |
| PrtMYB119-R | GCtctagaTTACAACATTATAGACTGAA | Use for the plasmid construction |
| PrtMYB119-F1 | AAGGCCATTACGGCCATGGTAGGCTCATTAGGAGTA | Use for the transactivation assay |
| PrtMYB119-R1 | CCGGCCGAGGCGGCCTTACAACATTATAGACTGAAGTCCT | Use for the transactivation assay |
| PrtMYB119-F2 | CTAAGGAAGTGCGTTGAGAA | Use for the qPCR |
| PrtMYB119-R2 | GCCAAGCAACTTGTGTAGTC | Use for the qPCR |

Supplementary Table S2. The sequences of the plasmid for pCAMBIA2300-*PtrMYB119* vector

**cacatacaaatggacgaacggataaaccttttcacgcccttttaaatatccgttattctaataaacgctcttttctctt
aggtttacccgccaatatatcctgtcaaacactgatagtttaaactgaaggcgggaaacgacaatctgatccaag
ctcaagctgctctagcattcgccattcaggctgcgcaactgttgggaagggcgatcggtgcgggcctcttcgctatt
acgccagctggcgaaagggggatgtgctgcaaggcgattaagttgggtaacgccagggttttcccagtcacgac
gttgtaaaacgacggccagtgccaagcttCATGGAGTCAAAGATTCAAATAGAGGACC
TAACAGAACTCGCCGTAAAGACTGGCGAACAGTTCATACAGAGTCTC
TTACGACTCAATGACAAGAAGAAAATCTTCGTCAACATGGTGGAGCA
CGACACACTTGTCTACTCCAAAAATATCAAAGATACAGTCTCAGAAGA
CCAAAGGGCAATTGAGACTTTTCAACAAAGGGTAATATCCGGAAACC
TCCTCGGATTCCATTGCCCAGCTATCTGTCACTTTATTGTGAAGATAG
TGGAAAAGGAAGGTGGCTCCTACAAATGCCATCATTGCGATAAAGGA
AAGGCCATCGTTGAAGATGCCTCTGCCGACAGTGGTCCCAAAGATGG
ACCCCCACCCACGAGGAGCATCGTGGAAAAAGAAGACGTTCCAACC
ACGTCTTCAAAGCAAGTGGATTGATGTGATATCTCCACTGACGTAAG
GGATGACGCACAATCCCACTATCCTTCGCAAGACCCTTCCTCTATATA
AGGAAGTTCATTTCATTTGGAGAGAACACGGGGGACTctgcaggtcgacAT
GGTAGGCTCATTAGGAGTAAGGAAAGGTGCATGGACGGAGGAGGAA
GATATACTTCTAAGGAAGTGCGTTGAGAAATATGGTGAAGGAAGATG
GCATGAAGTTCCTTCCAGAGCAGGCTTGAATCGATGCAGGAAAAGCT
GCAGAATGAGGTGGTTGAATTATCTTAAGCCAAATGTCAAGAGAGGA
CAGTTTTCGGTGGACGAAGTGGACTTGATTATCAGACTACACAAGTT
GCTTGGCAATAGGCAAGTGAAAATGTGGTCATTGATAGCTGGTAGAC
TTTCAGGAAGAACAGCGAATGATGTAAAGAATTATTGGAACTCAAAC
CAGCGTAAGAAGGTGATTTCTAGCACTGATGAAGTTCAATCAAAACC
AAAAGCAAAATCAATCACAAGAGACAACATAATAAAGCCTCAACCTTG
GAAGTTCAGAAATTTATTCTGGTTAAGAGGAAAAAGTACTCCACTTAT
TAATGTTGGTTCTCAATATGGGGACGATCTTTGTAAGCCATGTTATTC
AACAGTATCGCCACCTTCCGACATTAATGAAGTTGAAAGTATATGGTG
GGAAAGCTCGTTAGATGACAAAGAAATTAATCAAACGATCAACAGCA
GTTGTCTGGGTTCTGTTTCTGTTTCAGCAGCAGCAGCTTACCTAGAG
TCCAGCGAAAGTCATTTTGTAAAGAACAACGCACCAAGAGGGATAAA
AACTGGGGACGTGTTCTATGAACAAGGACAAAATTGTTGGAGTGACA
TTTCTTTGGATGCAGACCTTTGGAATCTAATCAATACAGAACTAGATC
AACAACAACCTGAAGGACTTCAGTCTATAATGTTGTAAtctagaGATCGT
TCAAACATTTGGCAATAAAGTTTCTTAAGATTGAATCCTGTTGCCGGT
CTTGCGATGATTATCATATAATTTCTGTTGAATTACGTTAAGCATGTAA
TAATTAACATGTAATGCATGACGTTATTTATGAGATGGGTTTTTATGAT
TAGAGTCCCGCAATTATACATTTAATACGCGATAGAAAACAAAATATAG
CGCGCAAACTAGGATAAATTATCGCGCGCGGTGTCaTCTATGTTACTA
GATCggatccccgggtaccgagctcgaattcgtaatcatggtcatagctgtttcctgtgtgaaattgttatccgct
cacaattccacacaacatacgagccggaagcataaagtgtaaagcctggggtgcctaatgagtgagctaactca
cattaattgcgttgcgctcactgcccgctttccagtcgggaaacctgtcgtgccagctgcattaatgaatcggccaa**

**cgcgcggggagaggcggtttgcgtattggctagagcagcttgccaacatggtggagcacgacactctcgtctact
ccaagaatatcaaagatacagtctcagaagaccaaagggctattgagacttttcaacaaagggtaatatcggga
aacctcctcggattccattgcccagctatctgtcacttcatcaaaaggacagtagaaaaggaaggtggcacctac
aaatgccatcattgcgataaaggaaaggctatcgttcaagatgcctctgccgacagtggtcccaaagatggaccc
ccacccacgaggagcatcgtggaaaaagaagacgttccaaccacgtcttcaaagcaagtggattgatgtgataa
catggtggagcacgacactctcgtctactccaagaatatcaaagatacagtctcagaagaccaaagggctattga
gacttttcaacaaagggtaatatcgggaaacctcctcggattccattgcccagctatctgtcacttcatcaaaagga
cagtagaaaaggaaggtggcacctacaaatgccatcattgcgataaaggaaaggctatcgttcaagatgcctct
gccgacagtggtcccaaagatggacccccacccacgaggagcatcgtggaaaaagaagacgttccaaccacgt
cttcaaagcaagtggattgatgtgatatctccactgacgtaagggatgacgcacaatcccactatccttcgcaaga
ccttcctctatataaggaagttcatttcatttggagaggacacgctgaaatcaccagtctctctctacaaatctatct
ctctcgagATGTTACGTCCTGTAGAAACCCCAACCCGTGAAATCAAAAAA
CTCGACGGCCTGTGGGCATTCAGTCTGGATCGCGAAAACTGTGGAAT
TGATCAGCGTTGGTGGGAAAGCGCGTTACAAGAAAGCCGGGCAATT
GCTGTGCCAGGCAGTTTTAACGATCAGTTCGCCGATGCAGATATTCGT
AATTATGCGGGCAACGTCTGGTATCAGCGCGAAGTCTTTATACCGAAA
GGTTGGGCAGGCCAGCGTATCGTGCTGCGTTTCGATGCGGTCACTCA
TTACGGCAAAGTGTGGGTCAATAATCAGGAAGTGATGGAGCATCAGG
GCGGCTATACGCCATTTGAAGCCGATGTCACGCCGTATGTTATTGCCG
GGAAAAGTGTACGTATCACCGTTTGTGTGAACAACGAACTGAACTGG
CAGACTATCCCGCCGGGAATGGTGATTACCGACGAAAACGGCAAGAA
AAAGCAGTCTTACTTCCATGATTTCTTTAACTATGCCGGAATCCATCG
CAGCGTAATGCTCTACACCACGCCGAACACCTGGGTGGACGATATCA
CCGTGGTGACGCATGTCGCGCAAGACTGTAACCACGCGTCTGTTGAC
TGGCAGGTGGTGGCCAATGGTGATGTCAGCGTTGAACTGCGTGATGC
GGATCAACAGGTGGTTGCAACTGGACAAGGCACTAGCGGGACTTTG
CAAGTGGTGAATCCGCACCTCTGGCAACCGGGTGAAGGTTATCTCTA
TGAACTGTGCGTCACAGCCAAAAGCCAGACAGAGTGTGATATCTACC
CGCTTCGCGTCGGCATCCGGTCAGTGGCAGTGAAGGGCCAACAGTT
CCTGATTAACCACAAACCGTTCTACTTTACTGGCTTTGGTCGTCATGA
AGATGCGGACTTACGTGGCAAAGGATTCGATAACGTGCTGATGGTGC
ACGACCACGCATTAATGGACTGGATTGGGGCCAACTCCTACCGTACC
TCGCATTACCCTTACGCTGAAGAGATGCTCGACTGGGCAGATGAACA
TGGCATCGTGGTGATTGATGAAACTGCTGCTGTCGGCTTTAACCTCT
CTTTAGGCATTGGTTTCGAAGCGGGCAACAAGCCGAAAGAACTGTAC
AGCGAAGAGGCAGTCAACGGGGAAACTCAGCAAGCGCACTTACAGG
CGATTAAAGAGCTGATAGCGCGTGACAAAAACCACCCAAGCGTGGTG
ATGTGGAGTATTGCCAACGAACCGGATACCCGTCCGCAAGTGCACGG
GAATATTTCGCCACTGGCGGAAGCAACGCGTAAACTCGACCCGACGC
GTCCGATCACCTGCGTCAATGTAATGTTCTGCGACGCTCACACCGATA
CCATCAGCGATCTCTTTGATGTGCTGTGCCTGAACCGTTATTACGGAT
GGTATGTCCAAAGCGGCGATTTGGAAACGGCAGAGAAGGTACTGGA
AAAAGAACTTCTGGCCTGGCAGGAGAAACTGCATCAGCCGATTATCA
TCACCGAATACGGCGTGGATACGTTAGCCGGGCTGCACTCAATGTAC**

**ACCGACATGTGGAGTGAAGAGTATCAGTGTGCATGGCTGGATATGTA**
**TCACCGCGTCTTTGATCGCGTCAGCGCCGTCGTCGGTGAACAGGTAT**
**GGAATTTCGCCGATTTTGCGACCTCGCAAGGCATATTGCGCGTTGGC**
**GGTAACAAGAAAGGGATCTTCACTCGCGACCGCAAACCGAAGTCGG**
**CGGCTTTTCTGCTGCAAAAACGCTGGACTGGCATGAACTTCGGTGAA**
**AAACCGCGCAGGGAGGCAAACAATGAATCAACAACTCTCCTGGCGC**
**ACCATCGTCGGCTACAGCCTCGGGAATTGCTACCGAGCTCGAGCTTG**
**GATGGATTGCACGCAGGgtSdTTCTCCGGCCGCTTGGGTGGAGAGGCT**
**ATTCGGCTATGACTGGGCACAACAGACAATCGGCTGCTCTGATGCCG**
**CCGTGTTCCGGCTGTCAGCGCAGGGGCGCCCGGTTCTTTTTGTCAAG**
**ACCGACCTGTCCGGTGCCCTGAATGAACTGCAGGACGAGGCAGCGC**
**GGCTATCGTGGCTGGCCACGACGGGCGTTCCTTGCGCAGCasTGTGC**
**TCGACGTTGTCACTGAAGCGGGAAGGGACTGGCTGCTATTGGGCGA**
**AGTGCCGGGGCAGGATCTCCTGTCATCTCACCTTGCTCCTGCCGAGA**
**AAGTATCCATCATGGCTGATGCAATGCGGCGGCTGCATACGCTTGATC**
**CGGCTACCTGCCCATTCGACCACCAAGCGAAACATCGCATCGAGCGA**
**GCACGTACTCGGATGGAAGCCGGTCTTGTCGATCAGGATGATCTGGA**
**CGAAGAGCATCAGGGGCTCGCGCCAGCCGAACTGTTCGCCAGGCTC**
**AAGGCGCGTATGCCCGACGGCGAGGATCTCGTCGTGACCCACGGCG**
**ATGCCTGCTTGCCGAATAdSgNTCATGGTGGAAAATGGCCGCTTTTCT**
**GGATTCATCGACTGTGGCCGGCTGGGTGTGGCGGACCGCTATCAGG**
**ACATAGCGTTGGCTACCCGTGATATTGCTGAAGAGCTTGGCGGCGAA**
**TGGGCTGACCGCTTCCTCGTGCTTTACGGTATCGCCGCTCCCGATTC**
**GCAGCGCATCGCCTTCTATCGCCTTCTTGACGAGTTCTTCTGActcgagtt**
**tctccataataatgtgtgagtagttcccagataagggaattagggttcctatagggtttcgctcatgtgttgagcata**
**taagaaacccttagtatgtatttgtatttgtaaaatacttctatcaataaaatttctaattcctaaaaccaaaatccag**
**tactaaaatccagatcccccgaattaattcggcgttaattcagtacattaaaaacgtccgcaatgtgttattaagttg**
**tctaagcgtcaatttgtttacaccacaatatatcctgccaccagccagccaacagctccccgaccggcagctcggc**
**acaaaatcaccactcgatacaggcagcccatcagtccgggacggcgtcagcgggagagccgttgtaaggcggc**
**agactttgctcatgttaccgatgctattcggaagaacggcaactaagctgccgggtttgaaacacggatgatctcg**
**cggagggtagcatgttgattgtaacgatgacagagcgttgctgcctgtgatcaccgcggtttcaaaatcggctccg**
**tcgatactatgttatacgccaactttgaaaacaactttgaaaaagctgttttctggtatttaaggttttagaatgcaa**
**ggaacagtgaattggagttcgtcttgttataattagcttcttggggtatctttaaatactgtagaaaagaggaagga**
**aataataaatggctaaaatgagaatatcaccggaattgaaaaaactgatcgaaaaataccgctgcgtaaaagat**
**acggaaggaatgtctcctgctaaggtatataagctggtgggagaaaatgaaaacctatatttaaaaatgacggac**
**agccggtataaagggaccacctatgatgtggaacgggaaaaggacatgatgctatggctggaaggaaagctgc**
**ctgttccaaaggtcctgcactttgaacggcatgatggctggagcaatctgctcatgagtgaggccgatggcgtcctt**
**tgctcggaagagtatgaagatgaacaaagccctgaaaagattatcgagctgtatgcggagtgcatcaggctcttt**
**cactccatcgacatatcggattgtccctatacgaatagcttagacagccgcttagccgaattggattacttactgaa**
**taacgatctggccgatgtggattgcgaaaactgggaagaagacactccatttaaagatccgcgcgagctgtatga**
**tttttaaagacggaaaagcccgaagaggaacttgtcttttcccacggcgacctgggagacagcaacatctttgtg**
**aaagatggcaaagtaagtggctttattgatcttgggagaagcggcagggcggacaagtggtatgacattgccttc**
**tgcgtccggtcgatcagggaggatatcggggaagaacagtatgtcgagctatttttgacttactggggatcaagc**
**ctgattgggagaaaataaaatattatattttactggatgaattgttttagtacctagaatgcatgaccaaaatccctt**

**aacgtgagttttcgttccactgagcgtcagaccccgtagaaaagatcaaaggatcttcttgagatcctttttttctgc
gcgtaatctgctgcttgcaaacaaaaaaaccaccgctaccagcggtggtttgtttgccggatcaagagctaccaa
ctctttttccgaaggtaactggcttcagcagagcgcagataccaaatactgtccttctagtgtagccgtagttaggc
caccacttcaagaactctgtagcaccgcctacatacctcgctctgctaatcctgttaccagtggctgctgccagtgg
cgataagtcgtgtcttaccgggttggactcaagacgatagttaccggataaggcgcagcggtcgggctgaacggg
gggttcgtgcacacagcccagcttggagcgaacgacctacaccgaactgagatacctacagcgtgagctatgag
aaagcgccacgcttcccgaagggagaaaggcggacaggtatccggtaagcggcagggtcggaacaggagagc
gcacgagggagcttccagggggaaacgcctggtatctttatagtcctgtcgggtttcgccacctctgacttgagcgt
cgatttttgtgatgctcgtcaggggggcggagcctatggaaaaacgccagcaacgcggcctttttacggttcctgg
ccttttgctggccttttgctcacatgttctttcctgcgttatcccctgattctgtggataaccgtattaccgcctttgagt
gagctgataccgctcgccgcagccgaacgaccgagcgcagcgagtcagtgagcgaggaagcggaagagcgcc
tgatgcggtattttctccttacgcatctgtgcggtatttcacaccgcatatggtgcactctcagtacaatctgctctga
tgccgcatagttaagccagtatacactccgctatcgctacgtgactgggtcatggctgcgccccgacacccgccaa
cacccgctgacgcgccctgacgggcttgtctgctcccggcatccgcttacagacaagctgtgaccgtctccgggag
ctgcatgtgtcagaggttttcaccgtcatcaccgaaacgcgcgaggcagggtgccttgatgtgggcgccggcggt
cgagtggcgacggcgcggcttgtccgcgccctggtagattgcctggccgtaggccagccatttttgagcggccag
cggccgcgataggccgacgcgaagcggcggggcgtagggagcgcagcgaccgaagggtaggcgctttttgcag
ctcttcggctgtgcgctggccagacagttatgcacaggccaggcgggttttaagagttttaataagttttaaagagt
tttaggcggaaaaatcgcctttttttctcttttatatcagtcacttacatgtgtgaccggttcccaatgtacggctttggg
ttcccaatgtacgggttccggttcccaatgtacggctttgggttcccaatgtacgtgctatccacaggaaagagacc
ttttcgacctttttcccctgctagggcaatttgccctagcatctgctccgtacattaggaaccggcggatgcttcgccc
tcgatcaggttgcggtagcgcatgactaggatcgggccagcctgccccgcctcctccttcaaatcgtactccggca
ggtcatttgacccgatcagcttgcgcacggtgaaacagaacttcttgaactctccggcgctgccactgcgttcgtag
atcgtcttgaacaaccatctggcttctgccttgcctgcggcgcggcgtgccaggcggtagagaaaacggccgatg
ccgggatcgatcaaaaagtaatcggggtgaaccgtcagcacgtccgggttcttgccttctgtgatctcgcggtaca
tccaatcagctagctcgatctcgatgtactccggccgcccggtttcgctctttacgatcttgtagcggctaatcaagg
cttcaccctcggataccgtcaccaggcggccgttcttggccttcttcgtacgctgcatggcaacgtgcgtggtgttta
accgaatgcaggtttctaccaggtcgtctttctgctttccgccatcggctcgccggcagaacttgagtacgtccgca
acgtgtggacggaacacgcggccgggcttgtctcccttcccttcccggtatcggttcatggattcggttagatggga
aaccgccatcagtaccaggtcgtaatcccacacactggccatgccggccggccctgcggaaacctctacgtgccc
gtctggaagctcgtagcggatcacctcgccagctcgtcggtcacgcttcgacagacggaaaacggccacgtccat
gatgctgcgactatcgcgggtgcccacgtcatagagcatcggaacgaaaaaatctggttgctcgtcgcccttggg
cggcttcctaatcgacggcgcaccggctgccggcggttgccgggattctttgcggattcgatcagcggccgcttgc
cacgattcaccggggcgtgcttctgcctcgatgcgttgccgctgggcggcctgcgcggccttcaacttctccaccag
gtcatcacccagcgccgcgccgatttgtaccgggccggatggtttgcgaccgtcacgccgattcctcgggcttggg
ggttccagtgccattgcagggccggcagacaacccagccgcttacgcctggccaaccgcccgttcctccacacat
ggggcattccacggcgtcggtgcctggttgttcttgattttccatgccgcctcctttagccgctaaaattcatctactc
atttattcatttgctcatttactctggtagctgcgcgatgtattcagatagcagctcggtaatggtcttgccttggcgta
ccgcgtacatcttcagcttggtgtgatcctccgccggcaactgaaagttgacccgcttcatggctggcgtgtctgcc
aggctggccaacgttgcagccttgctgctgcgtgcgctcggacggccggcacttagcgtgtttgtgcttttgctcatt
ttctctttacctcattaactcaaatgagttttgatttaatttcagcggccagcgcctggacctcgcgggcagcgtcgc
cctcgggttctgattcaagaacggttgtgccggcggcggcagtgcctgggtagctcacgcgctgcgtgatacggg
actcaagaatgggcagctcgtacccggccagcgcctcggcaacctcaccgccgatgcgcgtgcctttgatcgccc
gcgacacgacaaaggccgcttgtagccttccatccgtgacctcaatgcgctgcttaaccagctccaccaggtcggc**

**ggtggcccatatgtcgtaagggcttggctgcaccggaatcagcacgaagtcggctgccttgatcgcggacacagc
caagtccgccgcctggggcgctccgtcgatcactacgaagtcgcgccggccgatggccttcacgtcgcggtcaat
cgtcgggcggtcgatgccgacaacggttagcggttgatcttcccgcacggccgcccaatcgcgggcactgccctg
gggatcggaatcgactaacagaacatcggccccggcgagttgcagggcgcgggctagatgggttgcgatggtcg
tcttgcctgacccgcctttctggttaagtacagcgataaccttcatgcgttccccttgcgtatttgtttatttactcatcg
catcatatacgcagcgaccgcatgacgcaagctgttttactcaaatacacatcacctttttagacggcggcgctcg
gtttcttcagcggccaagctggccggccaggccgccagcttggcatcagacaaaccggccaggatttcatgcagc
cgcacggttgagacgtgcgcgggcggctcgaacacgtacccggccgcgatcatctccgcctcgatctcttcggta
atgaaaaacggttcgtcctggccgtcctggtgcggtttcatgcttgttcctcttggcgttcattctcggcggccgcca
gggcgtcggcctcggtcaatgcgtcctcacggaaggcaccgcgccgcctggcctcggtgggcgtcacttcctcgct
gcgctcaagtgcgcggtacagggtcgagcgatgcacgccaagcagtgcagccgcctctttcacggtgcggccttc
ctggtcgatcagctcgcgggcgtgcgcgatctgtgccggggtgagggtagggcgggggccaaacttcacgcctc
gggccttggcggcctcgcgcccgctccgggtgcggtcgatgattagggaacgctcgaactcggcaatgccggcg
aacacggtcaacaccatgcggccggccggcgtggtggtgtcggcccacggctctgccaggctacgcaggcccgc
gccggcctcctggatgcgctcggcaatgtccagtaggtcgcgggtgctgcgggccaggcggtctagcctggtcac
tgtcacaacgtcgccagggcgtaggtggtcaagcatcctggccagctccgggcggtcgcgcctggtgccggtgat
cttctcggaaaacagcttggtgcagccggccgcgtgcagttcggcccgttggttggtcaagtcctggtcgtcggtgc
tgacgcgggcatagcccagcaggccagcggcggcgctcttgttcatggcgtaatgtctccggttctagtcgcaagt
attctactttatgcgactaaaacacgcgacaagaaaacgccaggaaaagggcagggcggcagcctgtcgcgta
acttaggacttgtgcgacatgtcgttttcagaagacggctgcactgaacgtcagaagccgactgcactatagcagc
ggaggggttggatcaaagtactttgatcccgaggggaaccctgtggttggcatg**

Supplementary Table S3. Primers used in qRT-PCR analysis

| Primers | Sequence (from 5' to 3') |
|---|---|
| NtTubulin-F | AGATGTTCCGTCGTGTCAGTG |
| NtTubulin-R | TGCTTCCTCTTCATCCTCATATCC |
| NtSOD-F | ACCACCAGAAGCATCATCAGACT |
| NtSOD-R | TAATGTGACCTCCGCCGTTG |
| NtCAT-F | TTCTGCCCTTCTATTGTGGTTCC |
| NtCAT-R | ATGAGCACACTTTGGAGCATTAGC |
| NtADC1-F | GCTGGATTGCCTTCAGTTGC |
| NtADC1-R | TTCATACCCGCACCAAGACG |
| NtSAMDC-F | CCATCCTAAAGTTGGCTGAGACC |
| NtSAMDC-R | ACTGAGCACCAGGGAAAATGAA |
| NtERD10D-F | GGCGGGCAAAAGAAGACAGA |
| NtERD10D-R | GCCAAACAGCAGTAGCACAATCA |
| NtNCED3-1-F | CATTGCAGAACCATGGCCAAAA |
| NtNCED3-1-R | AGGCTCCCCACCAAACTTGT |
| NtNAC/RD26-F | GCTCCCAAATGGGACGACCT |
| NtNAC/RD26-R | GCATTTGGGGCTCAAAAGGGT |

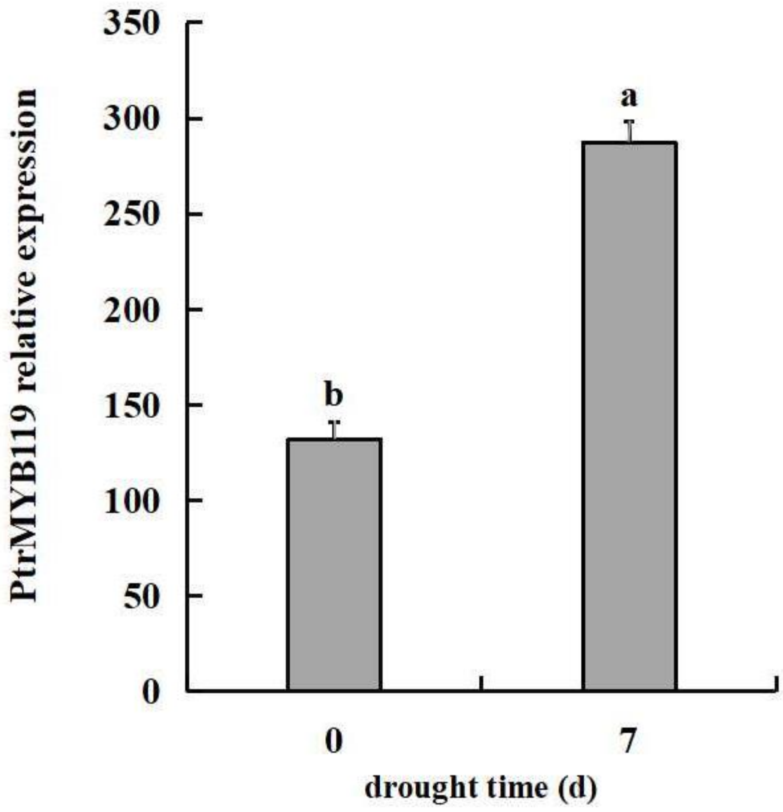


Supplementary Figure S1. Expression level of *PtrMYB119* in poplar after 0 and 7 days of drought treatment. Data are means ± SE of three biological replicates and means followed by different letters are significantly different ($P < 0.05$). The X-axis is the days of drought treatment.

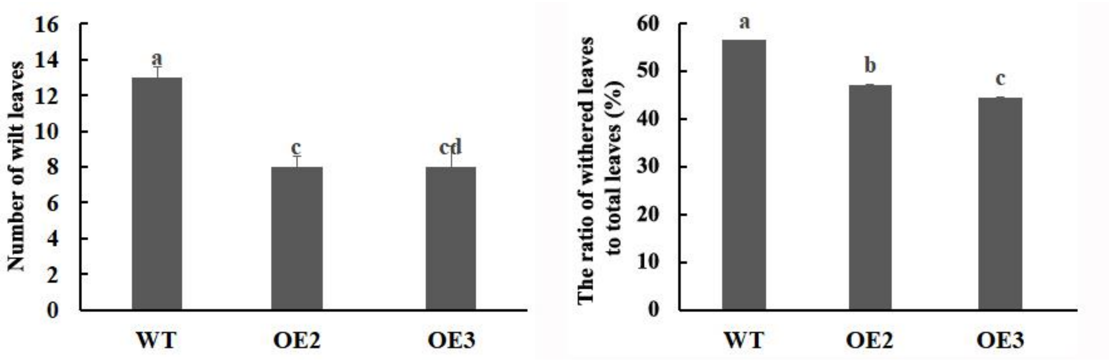


Supplementary Figure S2. Numbers of wilt leaves and ratio of withered leaves to total leaves in transgenic plants and WT plants after 30 days of drought treatment. Data are means ± SE of three biological replicates and means followed by different letters are significantly different ($P < 0.05$).

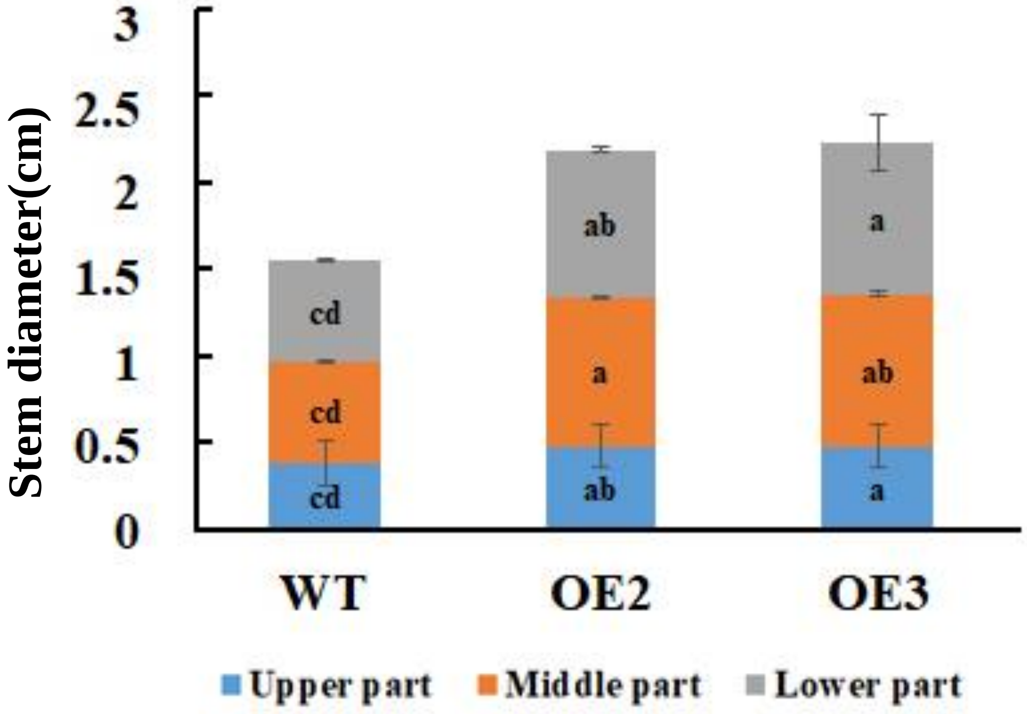


Supplementary Figure S3. Stem diameter of transgenic plants and WT plants after 30 days of drought treatment. Data are means ± SE of three biological replicates and means followed by different letters are significantly different ($P < 0.05$).

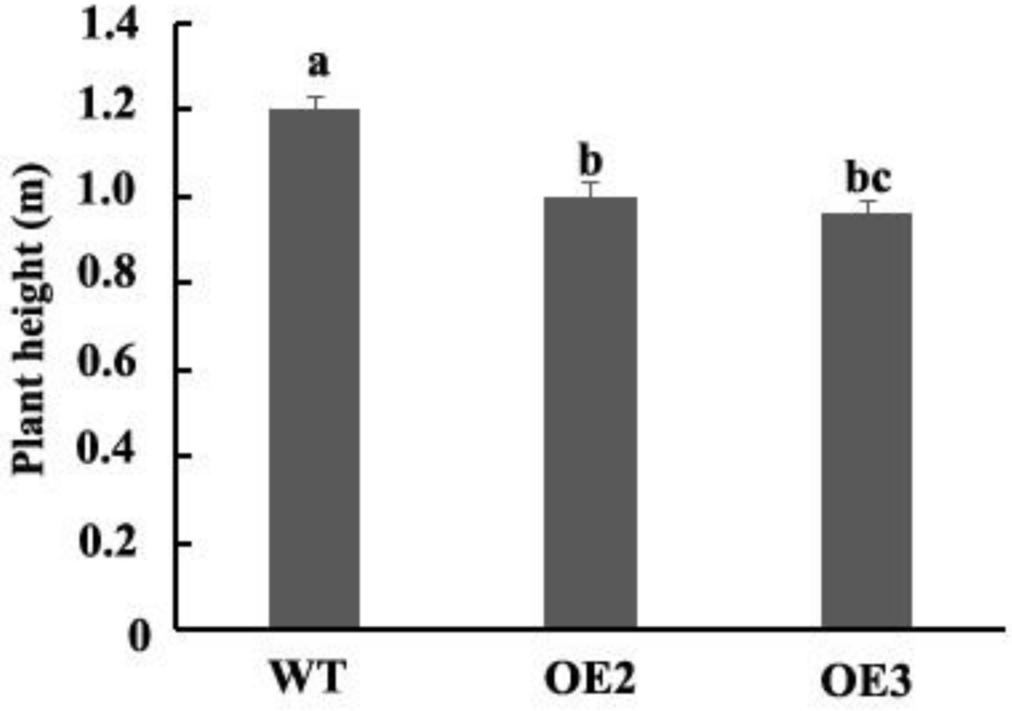


Supplementary Figure S4. Plant height of transgenic plants and WT plants after 30 days of drought treatment. Data are means ± SE of three biological replicates and means followed by different letters are significantly different ($P < 0.05$).

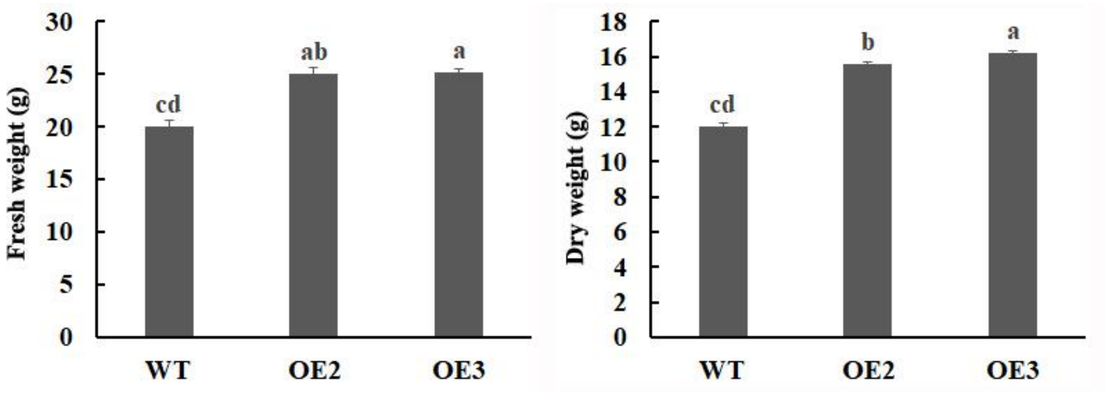


Supplementary Figure S5. Fresh weight and dry weight of transgenic plants and WT plants after 30 days of drought treatment. Data are means ± SE of three biological replicates and means followed by different letters are significantly different ($P < 0.05$).
